# Supplementary figures and images for: Imaging dendritic spines in the hippocampus of a living mouse by 3D-stimulated emission depletion microscopy
Source: Neurophotonics. 2023 May 17;10(4):044402. doi: 10.1117/1.NPh.10.4.044402 (PMC10197143; doi:10.1117/1.NPh.10.4.044402)

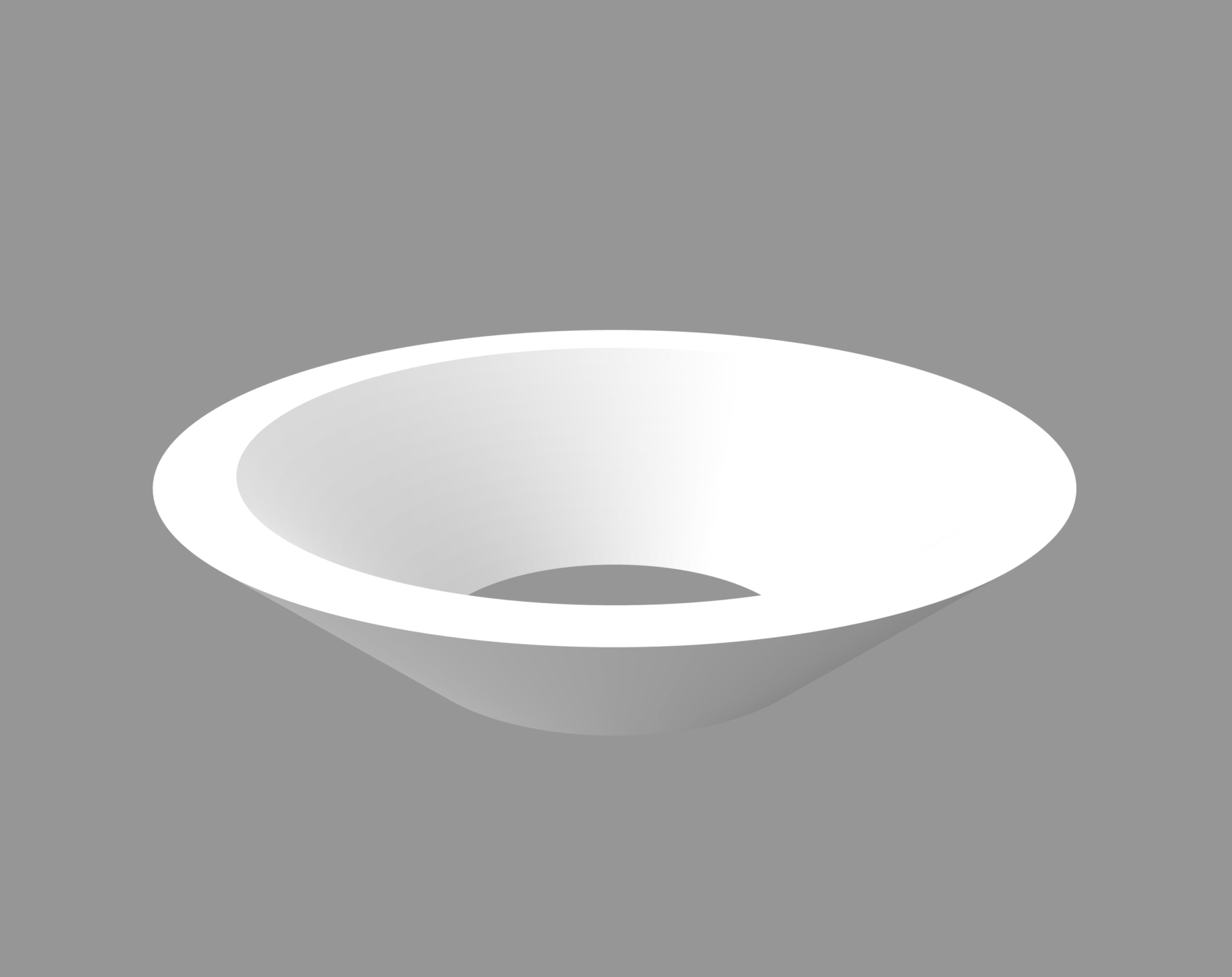

Supplement: Supplementary file 1 [file NPh_010_044402_SD001.jpg]
